# The Income Tax and Eugenics

**Published:** 1914-03

**Authors:** 


### The Income Tax and Eugenics.

The income tax features of the new tariff law do not encourage matrimony for the purpose of thrift, nor do they discourage race suicide. Living apart a man and woman are entitled to an exemption of $6000 per annum—or even living together and unmarried; but marriage immediately reduces this exemption to $4000 a year, and the law takes no notice of any additional mouths that the marriage may be responsible for. It seems hardly right, either upon legal, moral or eugenic grounds that the family should be penalized. France places a bounty upon fruitful marriages—the United States penalizes it.—*The Lancet-Clinic,* October 11, 1913.

